# Quality by Design-Optimized Glycerosome-Enabled Nanosunscreen Gel of Rutin Hydrate

**DOI:** 10.3390/gels9090752

**Published:** 2023-09-15

**Authors:** Md. Shabbir Alam, Niha Sultana, Md. Abdur Rashid, Yahya Alhamhoom, Asad Ali, Ayesha Waheed, Mo. Suheb Ansari, Mohd. Aqil, Mohd Mujeeb

**Affiliations:** 1 Department of Pharmaceutics, School of Pharmaceutical Education and Research, Jamia Hamdard, New Delhi 110062, India; shabbirobra786@gmail.com (M.S.A.); nihasultana94@gmail.com (N.S.); asad97111@gmail.com (A.A.); ayesha_waheed11@yahoo.com (A.W.); mosuheb0101@gmail.com (M.S.A.); mmujeeb@jamiahamdard.ac.in (M.M.); 2 Department of Pharmaceutics, College of Pharmacy, King Khalid University, Abha 62223, Saudi Arabia; mdrashid@kku.edu.sa (M.A.R.); ysalhamhoom@kku.edu.sa (Y.A.)

**Keywords:** sunburn, rutin, antioxidants, glycerosome, carbopol-gel, DoE

## Abstract

Sunburn is caused by prolonged exposure to ultraviolet (UV) rays from the sun, resulting in redness of the skin as well as tenderness, swelling, and blistering issues. During the healing process, it can cause peeling, irritation, and some long-term effects, including premature aging, pigmentation, and a high risk of skin cancer. Rutin has antioxidant and anti-inflammatory effects, which could potentially reduce inflammation and soothe sunburned skin. The objective of the current proposal is to develop and create carbopol gel-encased glycerosomes for the treatment of sunburn. The Design of Expert (DoE) technique was used to optimize the proposed formulation and was subjected to various characterization parameters such as nanovesicles size, polydispersity index (PDI), surface charge, entrapment efficiency (EE), and surface morphology. The optimized rutin-loaded glycerosomes (opt-RUT-loaded-GMs) were further characterised for drug release, 2,2-Diphenyl-1-picrylhydrazyl (DPPH) assay, and confocal laser scanning microscopy (CLSM). The formulation showed sustained release, greater permeation into the skin, and good antioxidant activity. The dermatokinetic study of opt-RUT-loaded-GMs confirms that the Rutin hydrate had better retention in the epidermis as compared to the dermis, owing to its potential for long lasting protection after topical application. It was observed that the prepared formulation was stable, highly safe, and had good sun protection factor (SPF) values that could be used as a suitable option for topical drug administration to maximize the therapeutic efficacy of the drugs.

## 1. Introduction

The skin, being the largest and most exposed organ of the body, is also highly vulnerable to photodamage resulting from sunlight exposure and other environmental factors. Although it plays a vital role in the production of vitamin D and is beneficial in the treatment of conditions such as psoriasis, dermatitis, and jaundice, prolonged exposure to ultraviolet (UV) radiation poses significant hazards. Chronic UV radiation exposure can lead to various adverse effects, including sunburn, erythema, inflammation, photoaging, and increased radiation levels. Among the different types of UV rays, ultraviolet-A (UV-A 320–400 nm) and ultraviolet-B (UV-B 290–320 nm) rays are particularly responsible for causing skin damage. While the ozone layer provides protection against the most harmful ultraviolet-C (UV-C 100–280 nm) rays, UV-B (over 5%) and UVA (approximately 90–95%) radiation penetrate through to the Earth’s surface, thereby impacting ecosystems and human well-being. Consequently, exposure to UV radiation can exert a significant influence on human health [1].

**Figure 1:**
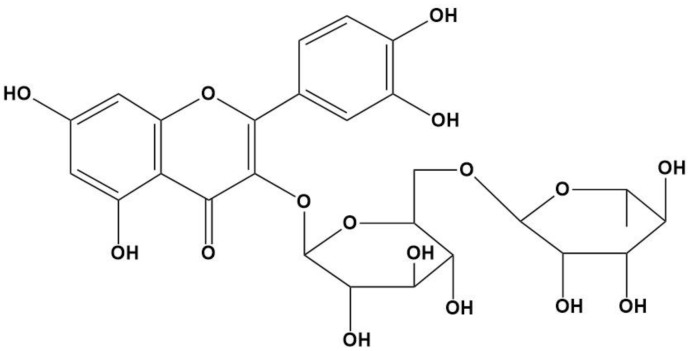
Chemical structure of Rutin.

As the main cause of suntan, erythema, and sunburn, UV-B radiation poses the greatest damage to human skin. The DNA of keratinocytes absorbs UV-B radiation when applied to human skin. Products containing sunscreen are used to protect against UV-A and UV-B radiation. Currently, sunscreens employ a combination of absorption, scattering, and reflection mechanisms to counteract UV energy. They incorporate both physically and chemically based agents for effective sun protection. Physical sunblocks contain substantial concentrations of zinc oxide (ZnO) and titanium dioxide (TiO_2_), which efficiently absorb UV-A and UV-B rays. However, frequent use of these chemicals can potentially harm the skin. Studies have shown that zinc oxide and titanium dioxide nanoparticles can generate reactive oxygen species (ROS), leading to oxidative DNA damage. As a result, there is growing interest in evaluating the antioxidant activity of plant-based products and employing natural antioxidants in skincare and cosmetics. Herbal extracts such as Rutin (RUT) (Figure 1) containing vitamins, alkaloids, flavonoids, phenolic acids, and terpenoids hold promise as potential ingredients in various pharmaceutical products aimed at enhancing skin health, including sunscreens. The development of a natural antioxidant is crucial to effectively protect the skin, as the primary factor contributing to the destruction of skin cells following UV exposure is the generation of reactive oxygen species [2].

Glycerosomes (GMs) represent an innovative approach to modify the fluidity of liposome bilayers through their elevated glycerol content, thereby enhancing their efficacy as a topical drug delivery system. GMs consist of various phospholipids along with 20–30% (*v*/*v*) non-toxic glycerol. These flexible vesicular carriers can incorporate diverse components, including cholesterol, which enhances the stability of the lipid bilayer. Furthermore, basic or acidic lipid molecules can be included to modulate the electrical charge of the vesicular surfaces and minimize vesicle aggregation. The production of GMs can be achieved using similar techniques employed for conventional liposomes. The presence of glycerol in these vesicles increases the compactness index, thereby augmenting therapeutic skin permeability and penetration. Cholesterol, acting as a barrier within the aqueous phase, improves GMs stability and preserves the integrity of the lipid membrane. Upon dispersion in the aqueous phase, phospholipids spontaneously form bilayer vesicles [3]. In the present study, a RUT-loaded GMs formulation was prepared and optimized utilizing the box behnken design (BBD). As BBD has the advantage of requiring a smaller number of runs compared to central composite, three-level full factorial designs, owing to three specific factors. In the context of full factorial designs, it is observed that as the number of factors increases, the number of trial runs also increases exponentially. For instance, when there are three factors, the total number of trial runs is 27. However, by utilizing the Box–Behnken design, it is possible to achieve optimization with only 17 experiments, including 5 center points [4].

## 2. Results and Discussion

### 2.1. Using the Quality by Design Method to Develop a Robust Formulation

**Figure 2:**
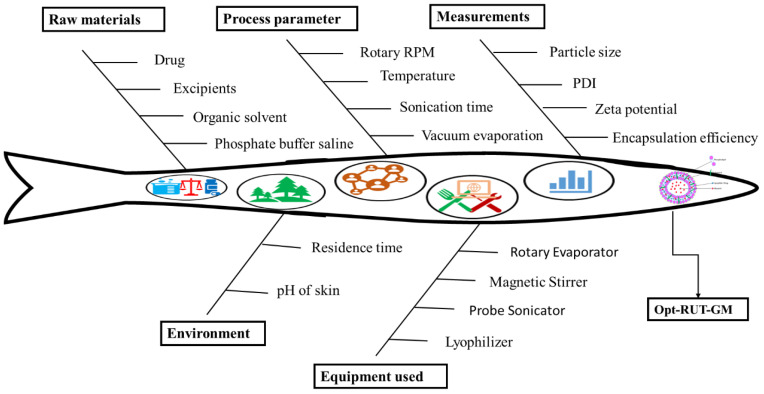
Ishikawa fishbone diagram.

QbD is an essential initial step to procure a suitable and reliable formulation. Table 1 lists many QTPP and CQA parameters that are derived from QTPP. The process of risk assessment was executed through the utilization of an Ishikawa fishbone diagram. This approach facilitated the examination of the impact of individual variables on critical quality attributes (CQAs), as illustrated in Figure 2.

### 2.2. Model Fitting and Optimization Using Box Behnken Design

The RUT-loaded-GM formulation was optimized using the BBD Expert program. A total of 17 formulations were created through the software’s 3-level and 3-factor experimental design (Table S1). The resulting polynomial equation represents various models, including the linear and quadratic effects of the independent variables on the dependent variables, as depicted in Figure S1. The equation’s negative and positive signs represented the positive and negative effects on the size of the vesicle and the entrapment efficiency. To assess the effect, the effects of the independent variables were further fitted to various kinetic models, including linear, 2 F1, and quadratic. The quadratic model was identified as the best among the several models since it demonstrated both the individual and combined effects of the variables on the dependent variables [9].

### 2.3. Optimization of RUT-Loaded GM by Statistical Design

The study was optimized and evaluated using the independent factors and their impacts on the response. Particularly, the amount of glycerol present in the formulation was key. Glycerol concentrations of 10% or lower resulted with reduced flexibility in vesicles, rapid deformation, and potentially limited penetration of skin. However, the flexibility of the vesicles increased as the formulation’s glycerol content increased. Notably, at 30% glycerol content, the GM exhibited significantly enhanced flexibility and resistance to the applied force. Glycerol may function as an active component in the phospholipid bilayer [10].

Furthermore, the inclusion of cholesterol in the GM composition contributed to the long-term stability of the phospholipids, augmented positive charges, and prevented vesicle agglomeration [11]. This addition of cholesterol enhanced the overall performance of the formulation. The summarized results of the experimental runs obtained through the Box–Behnken design are presented in Table S1, providing valuable insights into the study outcomes. The dependent variables group and quadratic models were found to be the most appropriate models. The optimal values of three dependent responses, vesicle size, polydispersity index, and entrapment efficiency, were determined using desirability criteria.

#### 2.3.1. Analysis of Response Surfaces

##### Impact of Independent Factor on Vesicle Size (Y_1_)

A quadratic polynomial equation aims to explain the effect of the concentration of phospholipid, cholesterol, and glycerol on vesicle size, as shown in Table S2**.** The size of the GM vesicles was significantly influenced favorably by the phospholipid concentrations. The developed formulation’s vesicle size analyses indicated a size range of 112.15 ± 4.03 nm to 186.16 ± 9.04 nm. The vesicle size at low phospholipid concentration (40 mg) was found to be 112.15 ± 4.03 nm (F17), and when the phospholipid concentration was increased to 50 mg, a significant increase in vesicle size was found to be at 149.95 ± 5.01 nm (F8). Whereas the increase in phospholipid concentration to 60 mg resulted in an increase in vesicle size to 186.16 ± 9.04 nm (F3) as shown in Table S1. The final result was comparable to the previously reported result [12]. The size of GM vesicles was favorably impacted by the cholesterol concentration. Vesicle size increased as cholesterol concentration rose from 2.5 to 7.5% *w*/*v*.

The vesicle size was less positively affected by the glycerol concentration. The vesicle size increases when glycerol concentration rises from 1 to 3% as shown in Table S1. The reduction in vesicle size at the same glycerol concentration, may be the result of the combined action of cholesterol and phospholipid, as the content of aqueous glycerol increased, the viscous fluid of glycerol induced an increase in vesicle formation [13].

##### Influence of Independent Factor on PDI

The quadratic equation observed for polydispersity index is shown in Table S2. The model has been determined to be significant with model F value of 29,560.93. The “Lack of Fit F-value” 6.41 indicates that it was non-significant. The “Predicted R^2^” of 0.9996 and the “Adjusted R^2^” of 0.9999 had a respectable degree of correlation. PdI values for RUT-loaded-GMs prepared according to the experimental design ranged between 0.194 ± 0.003 and 0.395 ± 0.0006. These indications indicate a linear model for glycerosome polydispersity index analysis. All of the formulations had polydispersity index values that were less than 0.3, except for F16, which had a PDI value of 0.395 and demonstrated issues with a wider range size distribution and agglomeration. The effects of individual parameters on PdI were observed using the response Y_2_ coded quadratic equation. PdI was observed to rise significantly as the concentration of phospholipid 90G, cholesterol and glycerol continued to increase.

##### Influence of Independent Factor on EE%

The observed quadratic equation for % EE is displayed in Table S2. The model has been determined to be significant with model F value of 1512.66. The “Lack of Fit F-value” 4.0 indicates that it was non-significant. The “Predicted R^2^” of 0.9988 and the “Adjusted R^2^” of 0.9936 had a respectable degree of correlation. The range of entrapment efficiency for the optimized RUT-loaded-GMs formulation was found to be 97.18 ± 2.47% to 98.0 ± 1.30%. The concentration of phospholipid, cholesterol, and glycerol has a favorable effect on the %EE. It was observed that formulation entrapment was enhanced when phospholipid concentration increased from 40 to 60 mg, cholesterol concentration from 2% to 7.5% *w*/*v*, and glycerol concentration from 1 to 3% as seen in Table S1.

#### 2.3.2. Experimental Design Validation

The consistency between predicted and experimental values is utilized to evaluate the validity of the optimization strategy. predicted values of the individual runs are presented in Table S1. The optimal formulation’s composition was established using the observed results from the responses. The software also produced the optimal formulation’s expected responses, which were compared to the optimized experimental formulation. In the BBD-optimized formula, 50 mg of phospholipid, 5 mg of cholesterol, and 2% of glycerol were included. The predicted values for vesicle size, PdI, and %EE of 149.43 nm, 0.296, and 83.78%, The were comparable with observed responses as 147.7 ± 1.85 nm, 0.295 ± 0.03, and 82.81 ± 3.85%, respectively. The findings showed a strong correlation between the predicted and actual values. As an outcome, opt-RUT-loaded-GMs developed with the optimal composition were used for further research.

#### 2.3.3. Optimized Level of Formulation by Point Prediction

The Design Expert software point prediction approach, the appropriate formulation of RUT-loaded-GMs was chosen on the basis of parameters of maximum entrapmentent, desirable vesicle size, and PDI. The optimized formulation has a vesicle size of 147.7 ± 1.85 nm, an entrapment efficiency 82.81 ± 3.85%, and a PDI of 0.295 ± 0.03. The resultant parameters are in accordance with the predicted values i.e., vesicle size, entrapment efficiency, and PDI to be 149.643, 83.478%, and 0.296, respectively, as generated by QbD.

### 2.4. Characterization of Opt-RUT-Loaded-GMs

#### 2.4.1. Determination of Vesicles Size, PDI, Zeta Potential and EE%

**Figure 3:**
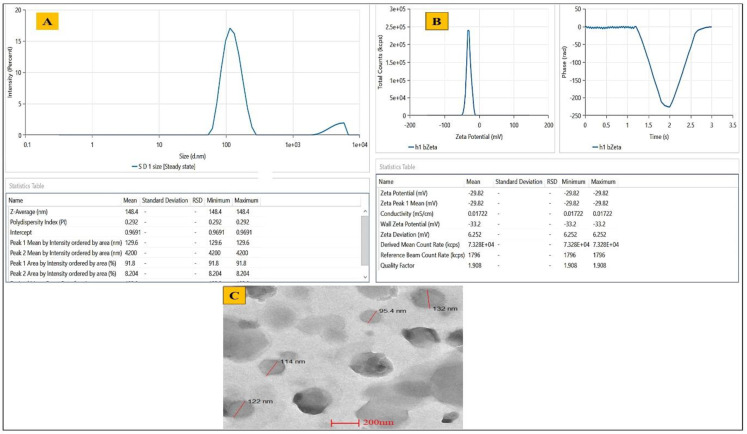
(**A**) vesicle size and (**B**) zeta potential of Opt-RUT-loaded-GMs and (**C**) TEM image of Opt-RUT-loaded-GM.

The opt-RUT-loaded-GMs formulation was observed to have a mean vesicle size of 123.7 ± 7.85 nm, and PDI value of 0.255 ± 0.04 (Figure 3A). The analyzed zeta potential of opt-RUT-loaded-GMs was -29.82 ± 2.03 mV (Figure 3B). An optimal stabilization of a nanodispersion is achieved when the value of zeta is more than -30 mV. The Zeta potential is a measurement employed to quantify the extent of the electrical charge present on the lipid bilayer and the surface charge of nanoparticles, which may be categorized as cationic, anionic, or neutral. The aforementioned technique has attained the status of a conventional method for the purpose of assessing and characterizing the surface of nanoparticles. A high zeta potential value, exhibiting both positive and negative charges, indicates enhanced stability of the nanoparticles. Based on the available evidence, it can be inferred that GMs exhibit a high degree of stability [14].

#### 2.4.2. Determination of Entrapment Efficiency

The opt-RUT-loaded-GMs entrapment efficiency was determined to be 82.81 ± 3.85%. The hydrophilic nature of rutin hydrate is a significant factor in entrapment, whereby an increase in phospholipid concentration can effectively enhance its entrapment into the lipid bilayer. Furthermore, elevation of the cholesterol concentration yields an augmented rigid bilayer architecture, thereby impeding the efflux of the drug from the GMs and ultimately leading to superior drug loading.

#### 2.4.3. Morphological Evaluation

The transmission electron microscopy (TEM) analysis of the opt-RUT-loaded-GMs indicates that the individual vesicles exhibit a smooth, uniform, and spherical morphology, with a diameter below 150 nm (Figure 3C). The confirmation of the nano size of the generated GMs is supported by the TEM images, thereby corroborating the results obtained from the Zetasizer particle size measurement.

#### 2.4.4. Differential Scanning Calorimetry

**Figure 4:**
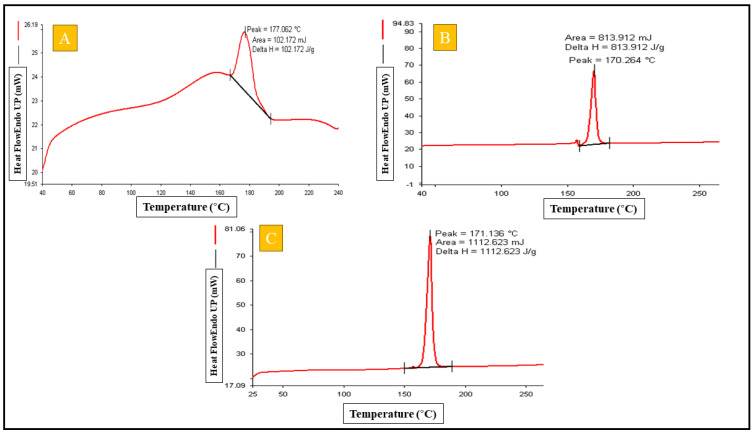
(**A**) DSC thermograms of Pure API-RUT, (**B**) Lyophilized Blank opt-GMs, (**C**) Lyophilized opt-RUT-loaded-GMs.

The DSC (Pyris 6 Perkin Elmer, USA) analysis of the rutin hydrate showed an extremely strong peak at 177.062 °C (Figure 4A) that verified the crystalline nature and the purity of the drug sample. The DSC thermogram for the blank opt-GMs formulation demonstrated only one peak (Figure 4B) at 170.264 °C which was found to be consistent with the peak of the cryoprotectant mannitol used to lyophilize the prepared GMs. Moreover, the opt-RUT-loaded-GMs indicated one peak (Figure 4C), which belonged to the cryoprotectant mannitol, at 171.1 °C. The findings indicated that there was no drug leakage or precipitate from the produced formulation of opt-RUT-loaded-GMs because no peak of the free drug was seen and the drug was completely encapsulated in GMs in an amorphous form.

#### 2.4.5. Compatibility Study Using Fourier Transform Infrared

FTIR spectra of Rutin hydrate showed bands in the region of 3409, 3322 (O-H stretching), 2901 (CH-stretching), 2173 (S-C≡N stretching), 1595 (N-H bending), 1651 (C-H bending), 1356 (S=O stretching), 1284 (C-N stretching). As depicted in Figure S2, the spectra of blank opt-GMs and opt-RUT-loaded-GMs formulations were found to be similar to that of RUT and no shift in the major peaks of RUT was detected, which further confirmed that there were no physiochemical interactions between excipients and drug (Figure S2) [15].

#### 2.4.6. Powder X-ray Diffraction Analysis

Rutin hydrate’s distinctive peaks may be seen in XRD spectra at the diffraction angles of 20.37°, 22.37°, and 26.11°. Consequently, the crystalline form of the drug was observed (Figure S3) [16]. This crystalline form was no longer present in the XRD spectra of opt-RUT-loaded-GMs as evidenced by the diminished characteristic peaks, demonstrating that the drug was completely distributed in a non-crystalline manner (Figure S3). The characteristic peaks of mannitol (20.43° and 21.39°) were also observed in an earlier report (Figure S3) [17].

#### 2.4.7. Antioxidant Analysis by 2,2-Diphenyl-1-picrylhydrazyl Assay Method

**Figure 5:**
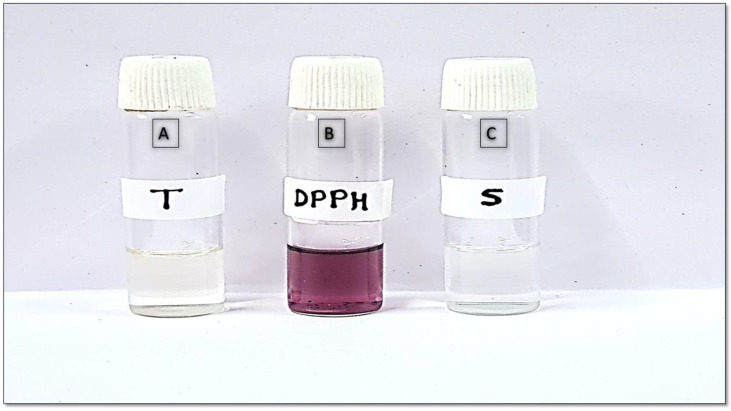
DPPH assay of (**A**) Rutin hydrate (after DPPH treatment), (**B**) Control, and (**C**) Ascorbic acid (after DPPH treatment).

The antioxidant potential of rutin hydrate was compared to that of a standard sample (ascorbic acid) Figure 5. The antioxidant activity of the rutin hydrate and the ascorbic acid were found to be 80.23 ± 1.563% and 87.21 ± 2.07%, respectively. Whereas the rutin hydrate and the ascorbic acid had IC50 values of 69.41 µg/mL and 65.61 µg/mL, respectively [18]. The study revealed an extra rise in the antioxidant efficacy of rutin hydrate in its pure form (80.23 ± 1.563% inhibition at 300 µg/mL) upon encapsulation in lipid-based vesicular system, viz. opt-RUT-loaded-GMs (86.82 ± 2.193%). An explanation for the increased antioxidant activity of RUT subsequent to entrapment within GMs may be attributed to the improved solubility and dissolution of RUT [19].

### 2.5. Characterization of Opt-RUT-Loaded-GMs Gel

The Opt-RUT-Loaded-GMs gel formulation had a homogeneous texture and appearance, was free of any discernible lumps, and had a smooth consistency. The pH of the gel formulation was observed in the range of 5.6 ± 0.15, whereas viscosity was found out to be 1566.667 ± 78.80 cp. The hardness, consistency, cohesiveness, and work of cohesion values for the gel were found to be 255.94 g and 291.64 g. s, 200.39 g, and 248.29 g. s, respectively (Figure S4). The extrudability and spreadability of gel was found to be 54.93 ± 2.40 and 15.32 ± 0.48. The results indicated that the prepared gel was appropriate for dermal application because it maintained adequate hardness, consistency, cohesiveness, and viscosity index.

### 2.6. In Vitro Drug Release Study

**Figure 6:**
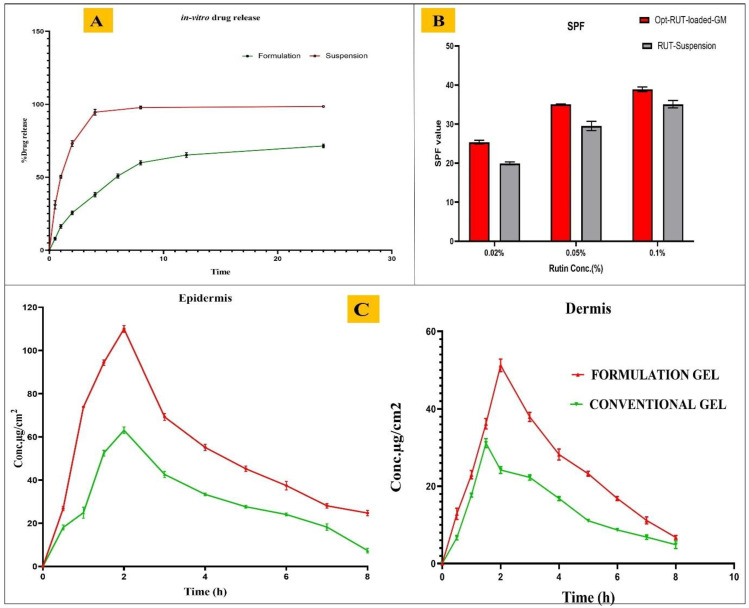
(**A**) In vitro release profile comparison of opt-RUT-loaded-GMs gel and RUT suspension, *p* < 0.0001, *n* = 3, (**B**) SPF value for Opt-RUT-loaded-GMs gel and RUT-Suspension at different concentrations (0.02%, 0.05% and 0.1%), *p* < 0.0001, *n* = 3. and (**C**) RUT concentration on the epidermis and dermis of excised rat skin following application to the skin of opt-RUT-loaded-GMs gel and RUT conventional gel, *p* < 0.05, *n* = 9.

The findings of an in vitro release study of opt-RUT-loaded-GMs gel versus RUT suspension are shown in Figure 6A. The drug was found to be released rapidly from the RUT suspension, with release of 95.46 ± 3.22% within 4 h of the study. Opt-RUT-loaded-GMs gel, on the other hand, demonstrated biphasic drug release. Initially, a burst release was detected in the first hour, which could be attributable to the unentrapped drug, whereas during the 24hr period, a total of 76.01 ± 2.24% of the drug was released.

The release kinetics were investigated by putting the data into various kinetic model equations. Table S3 provides data on release kinetics. The R^2^ value of the Higuchi model was found to be close to 1 and highest among all kinetic models confirming that it is best fit model. The results showed that the opt-RUT-loaded-GMs gel followed the Higuchi kinetic model (R^2^ = 0.9472), [20]. Whereas, according to the Korsemeyer–Peppas model, n exponent value was 0.0317, i.e., less than 0.45, indicating that the mechanism of drug release is a Fickian diffusion [21].

### 2.7. Sun Protection Factor (SPF) Evaluation

Four groups of SPF values are distinguished: non-sunscreen, low, medium, and high. If the score is less than 2, it is not in the category of sunscreens (non-sunscreen); if it is between 2 and 11, it offers only little protection (low SPF); if it is between 12 and 30, it offers medium protection (medium SPF); and if it is greater than or equal to 30, it offers strong protection (high SPF) [22]. The result showed that opt-RUT-loaded-GMs gel of 0.02% RUT concentration had medium protection, meanwhile, opt-RUT-loaded-GMs gel of 0.05% and 0.1% RUT concentration had high protection. On the other hand, RUT-Suspension of 0.02% RUT concentration had medium protection but slightly lower than that of opt-RUT-loaded-GMs gel (0.02%), similarly RUT-suspension of 0.05% and 0.1% RUT concentration showed high protection but less than that of opt-RUT-loaded-GMs gel (0.05% and 0.1%) as shown in Figure 6B. These SPF values were found to be good enough and proved that opt-RUT-loaded-GMs gel had greater effectiveness as a sun protector.

### 2.8. Confocal Laser Scanning Microscopy

**Figure 7:**
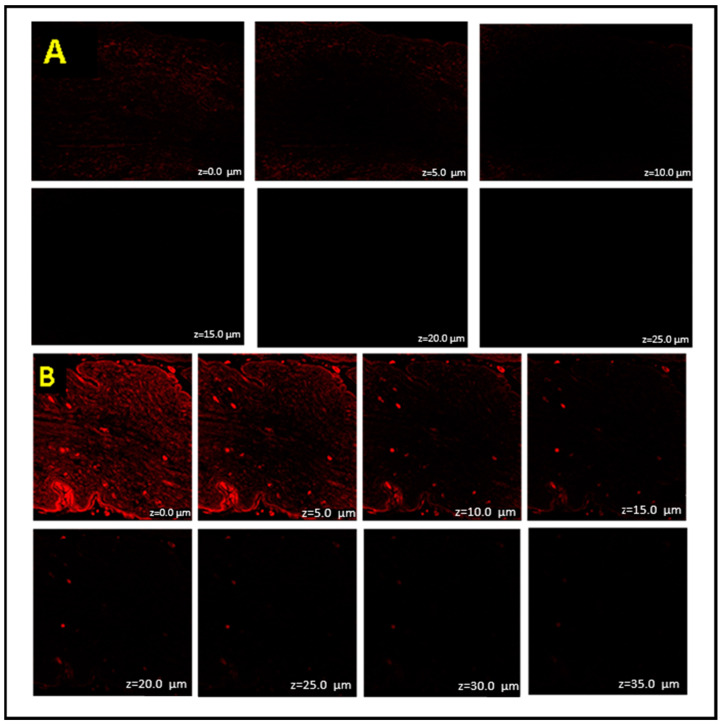
(**A**) CLSM of Rhodamine B dye hydroethanolic solution. (**B**) CLSM of opt-GMs loaded with Rhodamine B dye.

According to confocal laser scanning microscopy, the hydroalcoholic solution of Rhodamine B dye was only able to reach the skin’s outermost layers with a permeation upto 5 µm (Figure 7A). Rhodamine B dye loaded-GMs gel formulation, on the other hand, was well permeated across the skin, with observed permeation reaching more than 30 µm. (Figure 7B). It can be assumed that the GMs formulation passes this region and enters the deeper layers of the stratum corneum intact before releasing the drug through diffusion because the stratum corneum has a thickness of 10–40 µm as demonstrated by the increased fluorescence intensity in the upper region of the skin [23]. The composition of GMs also exerts an influence on the elevated permeation across the skin. Their smaller sizes and elasticity in altering the vesicular structure facilitate enhanced permeation into the skin layer.

### 2.9. Ex Vivo Dermatokinetic Study

According to the results of the dermatokinetic study, as shown in Figure 6C, a higher concentration of the drug was found in the epidermis and dermis of skin treated with opt-RUT-loaded-GMs gel as compared to RUT conventional gel. The enhanced drug absorption in the epidermal and dermis layers was demonstrated by the greater C_Skin max_ and AUC_0–8_ values observed in both layers upon application of the opt-RUT-loaded-GMs gel. This improvement can be attributed to the nanosized vesicles present in the gel, which facilitate penetration into the skin’s lipid layers. Several, dermatokinetic parameters of opt-RUT-loaded-GMs gel and RUT conventional gel are listed in Table 2 [24].

### 2.10. Skin Irritation Study

**Figure 8:**
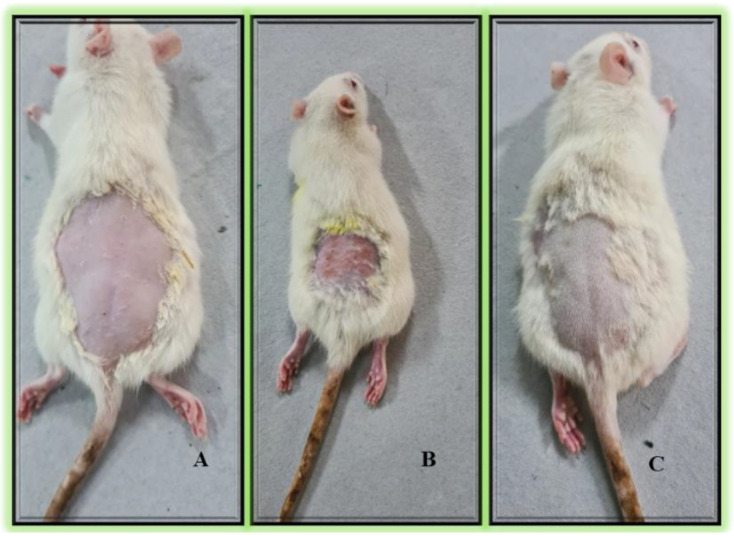
Skin irritation examination of rats (**A**) Negative control, (**B**) Positive control (0.8% formalin), and (**C**) opt-RUT-loaded-GMs gel formulation.

The results of the skin irritation test are displayed in Figure 8. The erythema values for negative control group and opt-RUT-loaded-GMs gel treatment group were 0 and 0.33 ± 0.57, respectively, whereas the edema scores were 0 as shown in Table 3. These results were observed to be significantly lower than the positive control (0.8% formalin) group, which had erythema and edema scores of 2.33 ± 0.57 and 1.33 ± 0.57, respectively. It was determined that the produced gel formulation was safe for use in topical application because it did not cause any skin irritation on the naked skin of Wistar rats as shown in Figure 8.

### 2.11. Accelerated Predictive Stability Study

Table 4 depicts the outcomes of stability experiments conducted on Opt-RUT-loaded-GMs liquid formulation and gel formulation that were preserved at temperatures of 4 ± 2 °C and 40 ± 2 °C/75 ± 5% RH. No significant alterations in phase separation or sedimentation were observed in glycerosomes when stored at a temperature of 4 °C, indicating their higher stability. The pH range of the formulation was observed to be between 5.7 ± 0.1 and 6.39 ± 0.13. The size of the vesicles in the formulation varied between 148.067 ± 0.75 nm and 184.1 ± 1.014 nm, while the PDI values ranged from 0.290 ± 0.004 to 0.35 ± 0.008. The study found that the entrapment efficiency was 83.25 ± 0.31% to 79.17 ± 0.36%, suggesting that the optimized formulation is expected to exhibit higher stability when stored at a temperature of 4 °C.

Opt-RUT-loaded-GMs display phase dispersion and particle aggregation, leading to an increase in sedimentation rate, following a storage period of three months at a temperature of 40 °C. At the conclusion of a three-month period, the vesicles of the GMs exhibited an increase in size to a measurement of 251.26 ± 2.31 nm. The phenomenon of improved particle sizes can be attributed to the conglomeration of vesicles, thereby elevating the likelihood of phase separation and sedimentation. The entrapment efficiency of drugs was reduced to 69.06 ± 0.448 due to the leakage from the GMs. Based on the empirical findings, it can be concluded that the optimal storage temperature for Opt-RUT-loaded-GMs is 4 °C. Whereas, opt-RUT-loaded-GMs gel formulation was found to be stable for 3 months at 4 ± 2 °C and 40 ± 2 °C/75 ± 5% RH.

## 3. Conclusions

A glycerosomal gel formulation containing rutin hydrate was effectively developed, optimized, and characterized. The predicted values in BBD exhibited a positive correlation with the observed values. The particle size of the formed Opt-RUT-loaded-GMs fell within the appropriate range for the intended use of topical delivery. The zeta potential analysis demonstrated that the formulation exhibited good stability. The incorporation of the API into the prepared Opt-RUT-loaded-GMs was confirmed by the results obtained from DSC and XRD studies. While the FTIR confirmed the API-excipient compatibility. Glycerosomes provided better penetration in comparison with the suspension. The Opt-RUT-loaded-GMs showed sustained release and good stability. Better penetration of glycerosomal gel than conventional gel was observed in a dermatokinetics study. Furthermore, the formulation was assessed for its potential in treating sunburn, which demonstrated effectiveness in treating sunburn, exhibiting a desirable sun protection factor (SPF) value and displaying favorable antioxidant properties. The formulation is determined to be suitable for safe administration via topical delivery as confirmed by a skin irritation study. Results confirmed that the formulated gel has the potential to maintain stability over prolonged periods when stored under normal conditions.

## 4. Material and Methods

### 4.1. Materials

Rutin Hydrate (RUT) was procured from Sigma-Aldrich, (Burlington, MA, USA), a product of China. Phospholipid 90G was procured from Lipoid, Ludwigshafen, Germany. Sisco Research Laboratories PVT LTD, Mumbai, India provided the cholesterol, DPPH, and rhodamine-B. Other chemicals like glycerol, HPLC Water, disodium hydrogen phosphate, potassium dihydrogen phosphate and HCL were purchased from SD Fine Chemicals, (Mumbai, India). Carbopol^®^ 934P NF (Carbopol 934P NF is used as Extended-release agent, Suspension and emulsion stabilizer and Bio adhesive agent) was received as a gift sample from Lubrizol Advanced Materials Inc., (Wickliffe, OH, USA). All the other solvents such as methanol, chloroform, ethanol, and octanol used were of laboratory grade, procured from Merck (Mumbai, India).

### 4.2. Methods

#### 4.2.1. Quality by Design (QbD) Identification of Component

A Quality Target Product Profile (QTPP) was developed to ensure that the final formulation meets the required standards for safety and efficacy, establishing the foundation for quality by design and ensuring the finished product’s desired performance [25]. The QTPP also encompasses Critical Quality Attributes (CQAs), which are derived from it and utilized to guide the development of products and processes. Moreover, CQAs define the range or limits for accepting high-quality products, ensuring the desired level of quality for the product. Differences in Critical Material Attributes (CMAs) and Critical Process Parameters (CPPs), which are associated with the composition of the formulation and variations in the critical manufacturing processes, respectively, are crucial in influencing CQAs. To assist in the assessment of risks, the guidelines of the international conference on harmonisation (ICH) Q9 have outlined various risk assessment tools [26]. Examples of such tools include Fault Tree Analysis, Failure Mode, and Effects Analysis (FMEA), and the Ishikawa Fishbone Diagram. In our study, an Ishikawa Fishbone Diagram was employed to establish the cause-effect relationship between different factors [27].

#### 4.2.2. Preparation of RUT-Glycerosomes (RUT-GMs)

The thin film hydration approach was employed for the preparation of RUT-loaded-GMs [28]. Cholesterol (5.0 mg/mL), phospholipid 90G (50.0 mg/mL), and RUT (5 mg/mL) were accurately measured and dissolved in chloroform with 1% methanol. The resulting mixture was mechanically stirred at 40 °C for one hour. Subsequently, the mixture was evaporated using a rotary evaporator (Hahnshin Scientific Co., Bucheon, Republic of Korea, HS-2005V-N) under reduced pressure, leading to the formation of a clear lipid film on the rounded bottom of the flask. The transparent lipid film allowed for the removal of residual solvents overnight under a vacuum. To hydrate the RUT-loaded-GMs, a glycerol-water solution (20% *w*/*v* glycerol) was used, and the two phases were mechanically stirred at 40 °C for one hour. The vesicles were then subjected to sonication for half cycle, which equated to 60 s, with a 3 s pause every 15 s. To remove any excess unentrapped drug, the resulting formulation was centrifuged at 7500 rpm for 10 min at 4 °C before being lyophilized for future use [3].

#### 4.2.3. Optimization of RUT-Loaded-GMs

The utilization of Design of Experiment (DOE) has been implemented in the development of glycerosomes. The optimization of RUT-loaded-GMs was conducted using the Box–Behnken design, which is a statistical experimental design technique that involves three factors with three levels each. The Design-Expert software (Version 12, Stat-Ease, MN, USA) was utilized for this purpose. The study aimed to optimize various process parameters, namely Phospholipid 90G (X1), cholesterol (X2), and Glycerol (X3), to assess their impact on the vesicle size (Y1), polydispersity index (Y2), and entrapment efficiency (Y3) of glycerosomes (Table S1). The independent variables were changed at low (−), medium (0), and high (+) concentrations in order to determine the optimal composition. The design illustrated a total of 17 formulation runs, each with distinct compositions, and included 5 center points to assess the impact of independent variables. The impact of independent variables was assessed through the examination of both the polynomial equation and response surface plot. The quadratic model was determined to be the optimal model due to its utilization of variables that demonstrated both individual and combined effects on the dependent variables.

#### 4.2.4. Characterization of Opt-RUT-Loaded-GMs

##### Vesicle Size, PDI and Zeta Potential

To measure the vesicle size, polydispersity index (PDI) and zeta potential the dynamic light scattering was employed using the Malvern Zetasizer, Nano ZS, Oxford, UK. Before analyzing the aforementioned parameters, the formulation was diluted 50 times approximately. The scattering angle was set at 90° and the temperature was held constant at about 25 ± 2 °C. Prior to experimentation, distilled water served as a control for the undiluted formulations. The study was conducted in triplicate to ensure reliability [28,29].

##### Entrapment Efficiency

Using the ultracentrifugation method, the entrapment effectiveness of the opt-RUT-loaded-GMs was assessed. A 10 mL glass centrifuge tube was filled with 2 mL of the formulation after the formulating glycerosomal preparation. The prepared formulation was diluted with distilled water to 5 mL and centrifuged (C-24 BL; Remi Instruments Ltd., Vasai, India) at 2000 rpm for 20 min while using a cooling centrifuge to separate the undissolved drug from the formulation. Ultracentrifugation was then performed for 30 min at 10,000 rpm to isolate the glycerosomes. The supernatant was collected from the pellets after centrifugation, and the amount of unentrapped drug was determined utilizing a UV spectrophotometer at a wavelength of 259 nm (Shimadzu-UV-1601). A UV calibration curve was generated for Rutin, which followed the formula: y = 0.0257x + 0.0358, where x represents absorbance and y represents rutin concentration. The coefficient of determination (R2) for the calibration curve was determined to be 0.99. Rutin content in glycerosomal formulation was determined by dividing the amount of drug entrapped (total drug added to formulation-free drug in supernatant) by the total amount of drug added during formulation preparation [30]. The below equation was used to calculate the entrapment efficiency.

(1) $$Entrapment\;efficinecy\%=\frac{total\;drug-free\;drug}{total\;drug}\times 100$$

##### Differential Scanning Calorimetery

The nature and entrapment of RUT was confirmed by performing Differential Scanning Calorimetery (Pyris 6 Perkin Elmer, Waltham, MA, USA) for drug samples and opt-RUT-loaded-GMs. A small portion of the sample was quickly sealed in an aluminum pan. The instrument was adjusted to a temperature range of 40 to 400 °C. The samples were heated at 10 °C/min using a nitrogen flow of 60 mL/min. 5% of mannitol was added as a cryoprotectant prior to freeze-drying the opt-RUT-loaded-GMs formulation for lyophilization [29].

##### Fourier Transform Infrared Compatibility Study

An FT-IR spectrometer was used to obtain FT-IR spectra (Bruker, Billerica, MA, USA). Potassium bromide was mixed separately with the pure API-RUT, blank opt-GMs formulation containing all excipients such as Phospholipon 90G, glycerine and cholesterol, and opt-RUT-loaded-GMs formulation (after lyophilization). The powders were compressed at a pressure of 15 tons for 10 min in a hydraulic press to create the potassium bromide discs and scanned from 4000 to 400 cm^−1^ [31].

##### X-ray Diffraction

An X-ray diffractometer (Pan Analytical, IIT Delhi, India) was used to assess the drug’s encapsulation with a scattering angle range of 20–80°. The diffractometer functioned at a voltage and current of 40 kV and 40 mA, respectively, and a rate of 0.6 min^−1^ was used to take the measurements. Rutin hydrate and opt-RUT-loaded-GMs that had been lyophilized had their XRD patterns identified utilizing the Origin software [16].

##### Surface Morphology of Opt-RUT-Loaded-GMs

Transmission electron microscopy (CRYO-TEM (TALOS S), Thermo Sceintific, Waltham, MA, USA.) was utilized to assess the surface morphology of the opt-RUT-loaded-GMs. A drop of prepared vesicular solution was placed on a carbon-copper coated grid and 1% phosphotungstic acid was used to negatively stain the sample. To prevent evaporation, the vesicular solution was applied to a carbon-coated TEM grid for 10 min in a moist environment, then, the surplus liquid was drained with filter paper. The prepared samples were observed using Transmission Electron Microscope and images for vesicular nanoparticles were captured [32].

##### Determination of Antioxidant Activity by 2,2-Diphenyl-1-picrylhydrazyl Assay

The standard 2,2-diphenyl-1-picrylhydrazyl (DPPH) technique was used to assess the antioxidant potential of RUT. The DPPH free radical method is widely used to evaluate a compound’s antioxidant potential. The electron-donating ability of antioxidants causes the violet-colored DPPH solution to turn colorless at room temperature. The standard in this assay was ascorbic acid (Merck, Mumbai, India). The sample (0.5 mL) was first dissolved in 3 mL of methanol before being treated with 0.3 mL of DPPH methanolic solution. The resulting reaction mixture was allowed to continue reacting for an additional hour in a dark environment. The color change indicates the sample’s antioxidant potential as a result of its hydrogen-donating ability. The blank contained 3.3 mL of methanol and 0.3 mL of sample, whereas the control contained 3.5 mL of methanol and DPPH solution (0.3 mL). At 517 nm, the sample was spectrophotometrically (Shimadzu-UV-1601) examined.

(2) $$Scavenging\;\%=\frac{Absorbance\;of\;control-Absorbance\;of\;sample}{Absorbance\;of\;control}\times 100$$

A graph showing the percentage of DPPH scavenging activity vs. different RUT concentrations was used to calculate the IC50 value for API (concentration at which it shows 50% of antioxidant activity) [9].

#### 4.2.5. Preparation of Opt-RUT-Loaded-GMs Carbopol^®^ Gel

The gelling ingredient carbopol^®^ 934P NF (Lubrizol Advanced Materials. Inc., Mumbai, India). was utilized for preparing gel formulations. The required carbopol^®^ powder (1%) was dissolved in distilled water and was kept overnight for proper mixing. The next day, 2–4 drops of triethanolamine were added to the carbopol^®^ mixture with continuous stirring, which led to the formation of a gel base due to the alteration in pH. Then, prepared opt-RUT-loaded-GMs were mixed with the gel base under continuous gentle stirring to prevent any bubble formation. Then, gel samples were continuously stirred for 60 min on a magnetic stirrer and opt-RUT-loaded-GMs Carbopol^®^ gel was obtained [33].

#### 4.2.6. Characterization of Opt-RUT-Loaded-GMs Gel

##### pH Evaluation

One gram of Opt-RUT-loaded-GMs gel formulation was weighed in a 50 mL volumetric flask and diluted with double distilled water and left for 2 h. The pH of the gel formulation was measured using a glass microelectrode (Mettler Instruments, Giessen, Germany) by keeping it in contact with the diluted gel for 1 min. Three distinct batches of gels were tested.

##### Homogeneity and Viscosity

All the gel formulations were transferred in transparent containers and were visually inspected for homogeneity. It was examined for the appearance of any type of aggregates in the gel formulation [34]. The viscosity of the prepared Opt-RUT-loaded-GMs gel was determined utilizing Brookfield viscometer using spindle no. 64 at 10 rpm and temperature of 25 °C [35].

##### Spreadability

The Opt-RUT-loaded-GMs gel spreadability was assessed utilizing the parallel plate technique. In this experimental protocol, a circular area with a diameter of 1 cm was marked on a glass plate, and 500 mg of the formulated gel was subsequently applied onto this area. Another glass plate was then placed on top of the gel. A duration of 5 min was allocated for the placement of a weight measuring 500 g onto the upper plate of the glass. The expansion of the diameter was observed as a result of the spreading of the gel [24]. The entire procedure was repeated three times (*n* = 3), and the average time of the three trials was used in subsequent calculations.

The following formula was used to determine the spreadability.

(3) $$S=\frac{M\times L}{T}$$

where L is the diameter of the spreaded gel, *S* is spreadability (g/s), M is the weight attached to top slides (gm), and *T* is time (s).

##### Extrudability

To evaluate the formulation’s extrudability, a closed, collapsible tube’s crimped end was forcibly squeezed utilizing weights. The formulation was extruded once the cap was taken off until the pressure subsided. The weight in grams required to extrude a 0.5 cm ribbon of the gel formulation in 10 s was calculated. The results were made in regard to the average extrusion pressure in grams i.e., *n* = 3 [36].

##### Texture Analysis

The gel was examined using a back extruding probe of 30 nm diameter in a texture analyzer (TA-XT2T Plus Texture Analyzer, StableMicro Systems Ltd., Surrey, UK). The consistency firmness, cohesiveness, consistency, and viscosity index were all determined using this technique [24].

#### 4.2.7. In Vitro Release

The in vitro release study of opt-RUT-loaded-GMs gel was carried out using a dialysis method. The pre-activated dialysis bag (MW-12 kDa) was utilized, both opt-RUT-loaded-GMs and RUT suspension was added to the bag following both the ends were tied using sealing clips. Phosphate buffer saline (pH 5.5, 250 mL) was used as dissolution media to immerse the dialysis bags in a 500 mL beaker. The beaker was then placed on a magnetic stirrer maintained at a temperature of 37.5 ± 0.5 °C and a moving speed of 100 rpm. A 1 mL sample aliquots were obtained at 0.5, 1, 2, 4, 6, 8, 12, and 24-h intervals. In order to equalize the sink conditions, the receiver compartment received 1 mL of fresh dissolution medium. The drug content of the withdrew samples were measured using UV spectrophotometer (Shimadzu-UV-1601) at 259 nm following dilutions [9,20]. The drug release kinetics of the formulation was accessed by fitting the data into different kinetic models (zero order, first order, Higuchi, and Korsmeyer–Peppas). The correlation coefficient (R2) for each model was calculated using the above-mentioned equations. Validated UV method in phosphate buffer (pH 5.5) was used for the quantification of drug released in the dissolution media having regression coefficient of 0.9743, limit of detection: 1.156 µg/mL, and limit of quantification: 3.503 µg/mL (*n* = 3).

#### 4.2.8. Sun Protection Factor Value Determination

The UV-VIS spectrophotometry was used to determine the effectiveness of RUT as sunscreen prepared into opt-RUT-loaded-GMs gel formulation and RUT suspension. The gel formulation and suspension were divided into different concentrations, F1 = 0.02%, F2 = 0.05%, and F3 = 0.1%. Each concentration was measured at intervals of 5 nm between 290 and 320 nm utilizing a UV-spectrophotometer (Shimadzu-UV-1601). Distilled water was utilized as a blank. The SPF value was determined using the absorbance value obtained for samples at different concentrations [22].

SPF = CF × $\sum_{320}^{290}EE$ (λ) × I (λ) × absorbance (λ)
CF = Correction factor (10)
EE = Erythmogenic effect of radiation with wavelength λ
I = Solar Ray Simulation Spectrum
Abs = spectrophotometric absorbance values at wavelength λ.

#### 4.2.9. Confocal Laser Scanning Microscopy

The study was performed on abdominal skin of rats to determine the penetration potential of GMs gel formulation. The opt-GMs gel formulation containing 0.02% *w*/*v* Rhodamine B dye and hydroethanolic solution of Rhodamine B dye was applied to rat skin in a non-occlusive manner fixed on Franz diffusion cell (donor chamber volume: 3 mL, receiver chamber volume: 10 mL, and a surface area of 1.76 cm^2^, manufactured by Rama Scientific, Delhi, India). The skin was maintained at 37.5 ± 0.5 °C with continuous stirring at 100 RPM for 8 h. The treated skin was taken out of the diffusion device after 8 h and rinsed with PBS solution to remove any leftover hydroalcoholic solution and adhering formulation. After being longitudinally cut with a microtome, the samples were mounted on glass slides. Confocal Lasser Scanning Microscopy (Leica Microsystem, TCS SPE; Milton Keynes, UK) was used to examine the fluorescent intensity of Rhodamine B dye on the slides. The sample was scanned through the z-axis using an Argon laser with excitation and emission wavelengths of 540 nm and 625 nm, respectively [17].

#### 4.2.10. Ex Vivo Dermatokinetics

The goal of the investigation was to determine the drug’s quantitative concentration in rat skin. A dermatokinetic study was conducted to determine the concentration of the drug in the epidermis and dermis layers of rat skin. The analysis was carried out by applying opt-RUT-loaded-GMs gel formulation and RUT suspension to prepared rat skin that had already been fixed on Franz diffusion cells (donor chamber volume: 3 mL, receiver chamber volume: 10 mL and a surface area of 1.76 cm^2^). After 0.5, 1, 1.5, 2, 3, 4, 5, 6, and 8 h, the entire skin sample was removed from the assembly. The formulation was adhered to the removed skin samples and was rinsed off three times with normal saline, and then the sample was immersed in warm water (60 °C) for 2–3 min. Following this, the epidermal and dermal layers of the rat’s skin were sliced separately, depauperated with 5 mL of methanol for 24 h at 37 °C, and the drug was completely extracted. The methanol solution was then filtered through a 0.25 µm pore size nylon syringe-driven filter, and the RUT content was determined in triplicate using UV spectroscopy (Shimadzu-UV-1601). Non-compartment dermatokinetics was used to calculate the concentration of RUT in each sample at each time point. PK solver software was used to estimate dermatokinetic parameters such as Cskin-max, AUC0–8 h, Ke, and Tskin-max [37]. Validated UV method in methanol was used for the quantification of drug concentration in different skin samples having regression coefficient of 0.9932, limit of detection: 1.310 µg/mL, and limit of quantification: 3.971 µg/mL (*n* = 3).

#### 4.2.11. In Vivo Study

The Central Animal House, Jamia Hamdard, New Delhi, India, vetted ethical compliance in animal handling. The approval for animal experimentation was granted by the Institutional Animal Ethics Committee (IAEC), Jamia Hamdard vide Protocol Number: 2055. The committee is registered with the Committee for the purpose of Control and Supervision of Experiments on Animals (CPCSEA) (173/GO/ReBi/S/2000/CCSEA 28 January 2000). The adult Wistar rats weighing in the range of 210–250 g were placed in polypropylene cages in the animal house adhering to IAEC and CPCSEA guidelines.

##### Skin Irritation Study

The Draize patch test was employed to investigate the potential irritation at the site of application. The study employed adult Wistar rats as experimental subjects. The dorsal region of the rats was shaved using a electrical clipper and razor. The rats were subjected to a 24-h period of observation to monitor for any injuries that may have been caused. The following day, a gel containing 0.5 g of opt-RUT-loaded-GMs was applied to the shaved area. The negative control group did not undergo any treatment, while the positive control group was treated with a 0.8% formalin solution. Erythema and edema were observed at various time intervals. The Draize scoring system was employed, utilizing a scale of 0 to 4, with 0 denoting the absence of erythema and edema, and 4 indicating the most severe manifestation of erythema and edema [38].

#### 4.2.12. Accelerated Stability Study According to ICH Q1A (R2) Guidelines

A stability investigation was performed on the optimized RUT-loaded GM liquid formulation and gel formulation to evaluate whether there will be any physical or chemical changes during storage. The formulation was stored for 3 months in a Borosil amber-colored glass container at 40 ± 2 °C, 75 ± 5%RH and 4 ± 0.5 °C for a stability assessment. Following storage, samples were obtained at 0-day, 1 week, 2 weeks, 1 month, 2 months, and 3 months intervals. Various parameters such vesicle size, PDI, % entrapment efficiency, physical appearance, color, phase separation, clarity, pH, homogeneity and washability were then used to determine the stability [39].

## Figures and Tables

**Table 1:** QTPP specifications and CQAs parameters for fabrication of opt-RUT-loaded-GMs.

QTPP	Target	Justification
Drug delivery system	Glycerosome	Improved skin permeability is preferred by glycerosomes.
Release type	Sustained release	Slow drug release is necessary to ensure sufficient drug concentration for proper sunburn action.
Route of administration	Topical	In comparison to oral treatment, it provides localized action with minimum systemic side effects.
Dosage form	Hydrogel	Provides easier topical application and improved penetration through the skin
Appearance	white gel with a smooth texture	It is free from gritty particles; the gel is uniformly smooth as well as odorless and Colorless.
Stability	>3 months	Maintains the therapeutic efficacy of the formulation.
CQAs	Target	Justification
Phospholipid 90G	Optimal vesicle size	Phospholipid 90G is a complex of phospholipids that contributes to the development of the phospholipid bilayer and also affects the size of the vesicles. [5]
Cholesterol	Fluidity and rigidity	Influence of Cholesterol on vesicle size and membrane fluidity. The formation of the phospholipid bilayer involves these components. [6]
Glycerol	Greater flexibility	The glycerol in these vesicles increases the deformability index, resulting in improved therapeutic skin penetration and permeability. [7]
Hydration media	Optimal glycerosome stability (absence of aggregation)	The lipid film is hydrated as part of the process of creating the phospholipid bilayer. [8]
API	The lipophilic API has been successfully incorporated into the glycerosomal wall.	The size of the vesicles may change depending on the nature of API included in the glycerosomes product [5].
Particle size	Less than 300 nm	To demonstrate enhanced drug penetration at the targeted skin layers with controlled drug release pattern.
Polydispersity index	Less than 0.5	Better homogeneity will result in more effective drug entrapment and release.
Entrapment Efficiency	More than 80%	Ensures high drug loading, and improved therapeutic results.

**Table 2:** Dermatokinetic parameters of opt-RUT-loaded-GMs gel and RUT conventional gel.

	Rutin Conventional Gel		Opt-RUT-Loaded-GMs Gel	
Dermatokinetic Parameters	Epidermis	Dermis	Epidermis	Dermis
T_Skin max_ (h)	2	1.5	2	2
C_Skin max_ (µg/cm^2^)	64.8814	32.4635	118.302	52.7329
AUC_0–8_ (µg/cm^2^ h)	248.3277	116.900	438.7941	197.4062
K_e_ (h^−1^)	0.15524	0.145283	0.129488	0.143148

**Table 3:** Skin irritation score of Wister rat treated with opt-RUT-loaded-GMs gel formulation.

Rats	Negative Control		Positive Control		RUT-Loaded-GM Gel	
S no.	Erythema	Edema	Erythema	Edema	Erythema	Edema
1	0	0	2	1	0	0
2	0	0	2	1	0	0
3	0	0	3	2	1	0
-	0	0	2.33 ± 0.57	1.33 ± 0.57	0.33 ± 0.57	0

Level of Erythema scale: 0, none; 1, minor; 2, well-defined; 3, moderate; 4, scar. Level of Edema scale: 0, none; 1, slight; 2, well-defined; 3, moderate; 4, severe.

**Table 4:** Accelerated and Short-term accelerated stability study parameters for opt-RUT-loaded-GMs formulation.

Storage Time	Appearance	Separation	pH ± SD	Vesicle Size (nm ± SD)	EE (% ±SD)	PDI (±SD)
Storage condition (40 °C ± 2 °C)						
0 day	Clear	No	5.7 ± 0.1	148.067 ± 0.75	83.25 ± 0.31	0.290 ± 0.004
1 week	Clear	No	5.63 ± 0.20	150.36 ± 1.33	84.14 ± 0.88	0.29 ± 0.001
2 weeks	Clear	No	5.94 ± 0.17	156.4 ± 0.79	83.34 ± 0.26	0.32 ± 0.01
1 month	Clear	No	5.99 ± 0.11	163.67 ± 1.86	82.14 ± 0.54	0.319± 0.003
2 months	Turbid	No	6.26 ± 0.06	172.8 ± 1.11	80.82 ± 0.7	0.382 ± 0.064
3 months	Turbid	Yes	6.39 ± 0.13	184.1 ± 1.014	79.17 ± 0.36	0.35 ± 0.008
Storage condition (40 ± 2 °C/75 ± 5% RH)						
0 day	Clear	No	5.76 ± 0.152	147.22 ± 1.03	82.90 ± 0.655	0.289 ± 0.004
1 week	Clear	No	5.69 ± 0.10	155.64 ± 0.89	81.24 ± 1.009	0.362 ± 0.014
2 weeks	Clear	No	6.16 ± 0.254	166.62 ± 0.79	81.82 ± 0.796	0.389 ± 0.009
1 month	Turbid	Yes	6.49 ± 0.26	188.51 ± 0.89	78.58 ± 0.94	0.45 ± 0.01
2 months	Milky	Yes	6.39 ± 0.142	226.33 ± 2.28	73.54 ± 1.36	0.55 ± 0.01
3 months	Milky	Yes	6.56 ± 0.210	251.26 ± 2.31	69.06 ± 0.448	0.64 ± 0.012
**Evaluation** **Parameters**	Initial		1 month		3 months	
			4 ± 2 °C	40 ± 2 °C/75 ± 5% RH	4 ± 2 °C	40 ± 2 °C/75 ± 5% RH
Color	Whitish		Whitish	Whitish	Whitish	Whitish
Appearance	Translucent		Translucent	Translucent	Translucent	Translucent
Phase Separation	None		None	None	None	None
Clarity	***		***	***	***	**
pH	5.65 ± 0.28		5.71 ± 0.16	5.8 ± 0.34	5.73 ± 0.12	5.87 ± 0.43
Homogeneity	***		***	***	***	***
Washability	Washable		Washable	Washable	Washable	Washable
Odor	None		None	None	None	None

* Satisfactory, ** Good, *** Excellent.

## Data Availability

Data will be available on request.

## Supplementary Materials

The following supporting information can be downloaded at: https://www.mdpi.com/article/10.3390/gels9090752/s1, Figure S1: Response surface plots showing the relative impacts of independent variables i.e., Phospho-lipid 90G conc. (mg); Cholesterol conc. (mg); and Glycerol conc. (% *w*/*v*). on (A) Vesicle size, (B) Polydispersity, and (C) % Entrapment efficiency; Figure S2: (A) FTIR spectra of the pure API- RUT, (B) Lyophilized Blank opt-GMs and (C) Lyophilized opt-RUT-loaded-GMs; Figure S3: (A) XRD spectrum of pure drug and (B) opt-RUT-loaded-GMs; Figure S4: Texture analysis report of opt-RUT-loaded-GMs gel formulation; Table S1: Responses observed in BBD for development and optimization of RUT-loaded-GMs formulations with a summary of parameters for responses- X1: Phospholipid 90G (mg), X2: Cholesterol (mg), X3: Glycerol (%*w*/*v*), Y1: Vesicle Size (nm), Y2: Polydispersity Index, Y3: Entrapment Efficiency (%); Table S2: Regression analysis response summary; Table S3: Regression coefficient of different release models for opt-RUT-loaded-GMs.
