# Supplementary material for: Quality by Design-Optimized Glycerosome-Enabled Nanosunscreen Gel of Rutin Hydrate
Source: Gels. 2023 Sep 15;9(9):752. doi: 10.3390/gels9090752 (PMC10531150; doi:10.3390/gels9090752)
Supplement: Supplementary file 1 [file gels-09-00752-s001.zip › gels-2543199-supplementary.pdf]

Supplementary

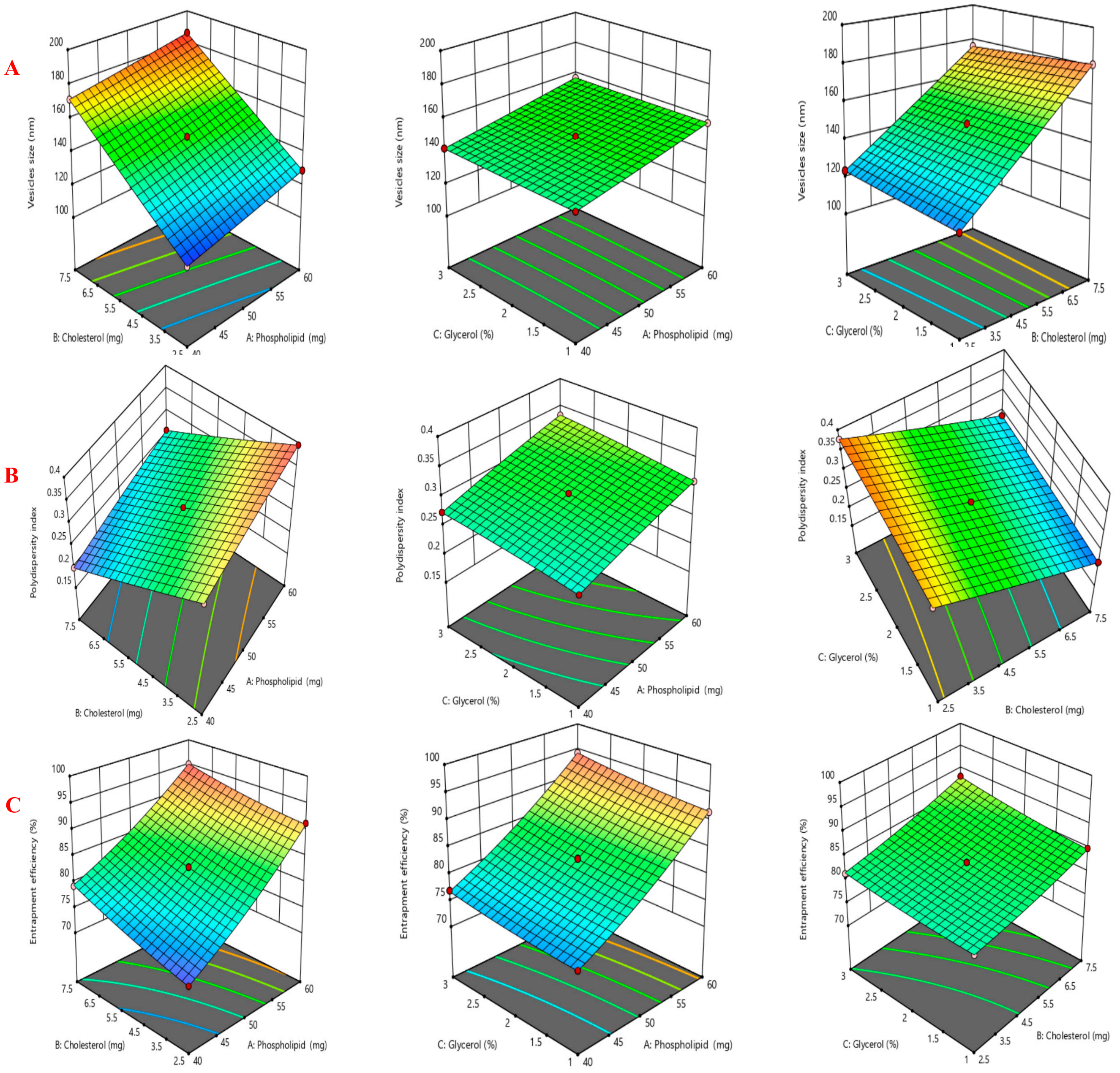

Figure S1: Response surface plots showing the relative impacts of independent variables i.e., Phospho-lipid 90G conc. (mg); Cholesterol conc. (mg); and Glycerol conc. (% w/v). on **(A)** Vesicle size, **(B)** Polydispersity, and **(C)** % Entrapment efficiency.

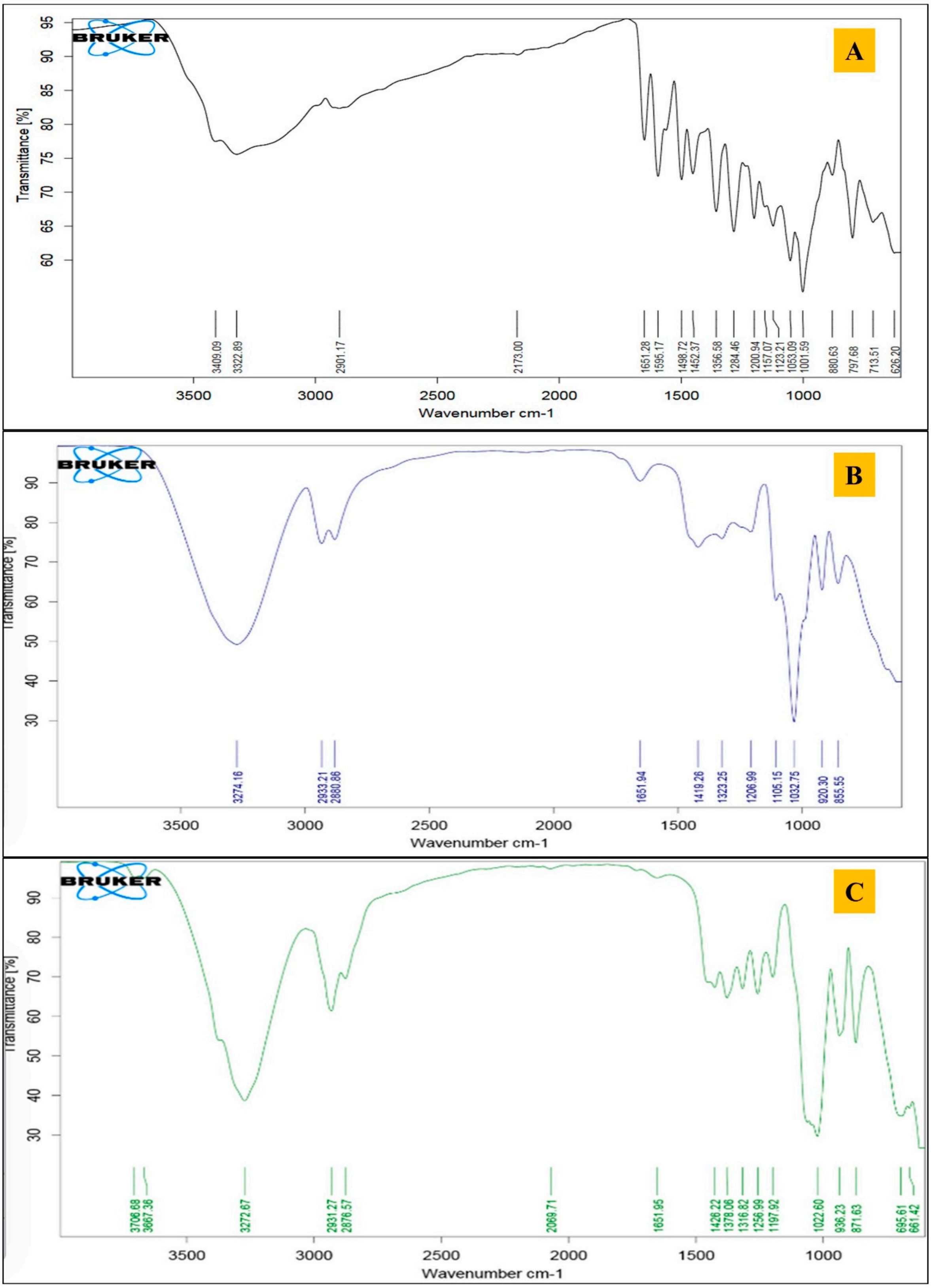

Figure S2: A- FTIR spectra of the pure API- RUT, **B-**Lyophilized Blank opt-GMs and **C-**Lyophilized opt-RUT-loaded-GMs.

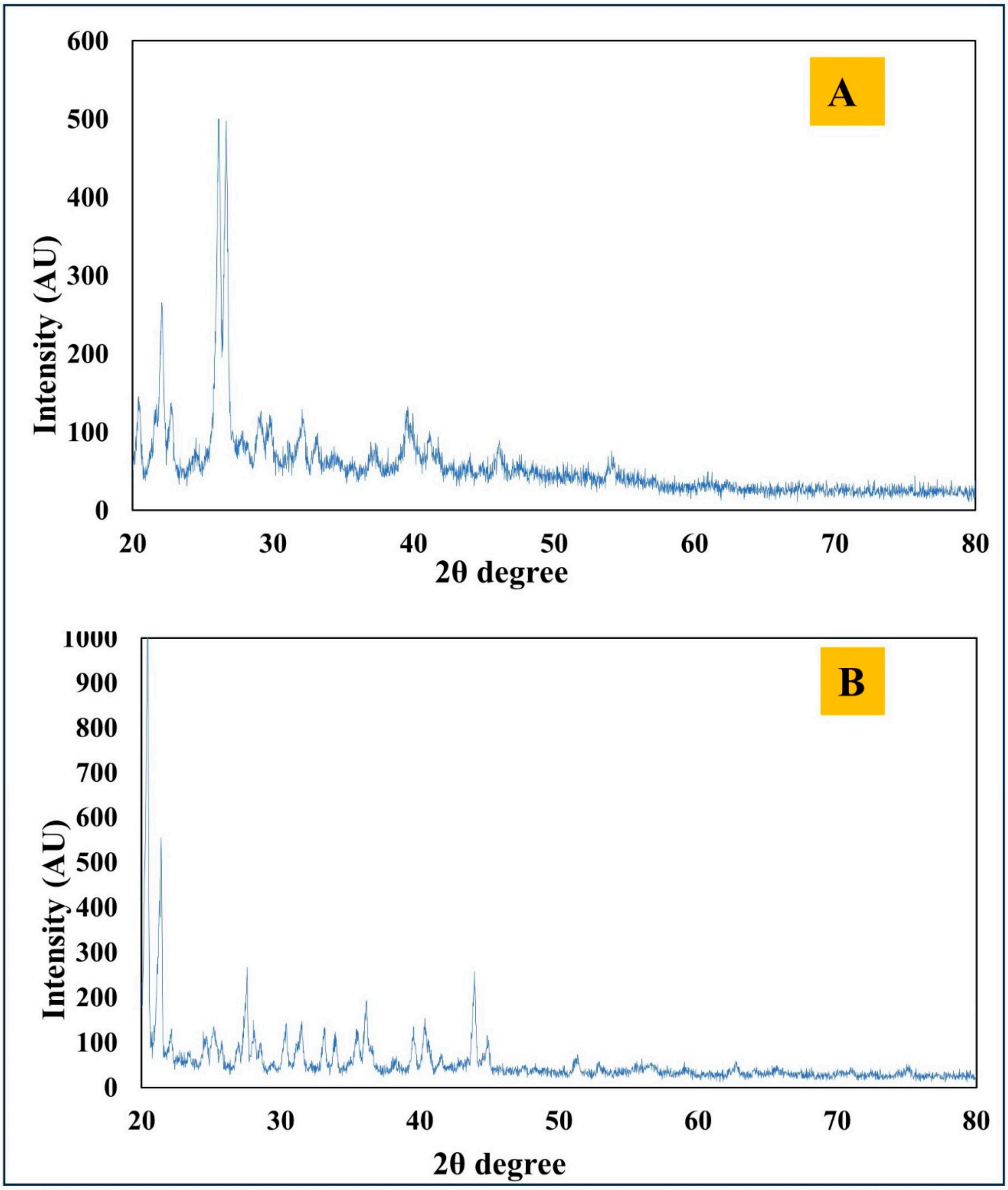

Figure S3: A- XRD spectrum of pure drug and **B-** opt-RUT-loaded-GMs.

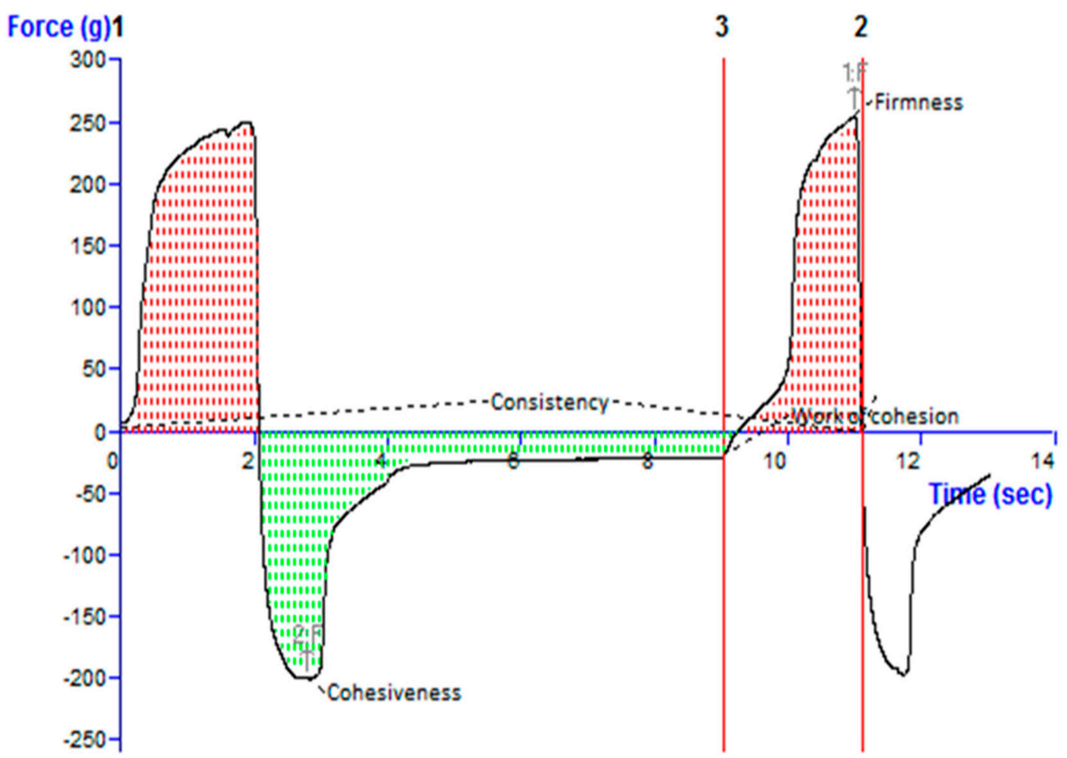

Figure S4: Texture analysis report of opt-RUT-loaded-GMs gel formulation

Table S1: Responses observed in BBD for development and optimization of RUT-loaded-GMs formulations with a summary of parameters for responses- X_1: Phospholipid 90G (mg), X_2: Cholesterol (mg), X_3: Glycerol (%w/v), Y_1: Vesicle Size (nm), Y_2: Polydispersity Index, Y_3: Entrapment Efficiency (%).

**Independent factors**				**Dependent factors**					
				**Observed values**			**Predicted values**		
**S.NO.**	**X_1**	**X_2**	**X_3**	**Y_1**	**Y_2**	**Y_3**	**Y_1**	**Y_2**	**Y_3**
F1	40	7.5	2	171.64 ± 3.59	0.1947±0.0030	79.13±0.53	171.31	0.1986	79.44
F2	50	5	2	149.20 ± 6.93	0.2907±0.0061	83.56±1.53	149.16	0.2977	82.84
F3	60	7.5	2	186.16 ± 9.04	0.2592±0.0036	95.34±0.98	185.91	0.2578	95.09
F4	50	7.5	3	176.26 ± 3.03	0.2378±0.0013	88.16±0.59	176.08	0.2392	88.23
F5	60	5	1	157.31 ± 5.94	0.3132±0.0023	91.40±3.17	157.49	0.3108	91.61
F6	50	5	2	149.57 ± 6.45	0.2971±0.0011	82.56±1.79	149.16	0.2977	82.84
F7	50	5	2	149.46 ± 6.47	0.2963±0.0016	82.61±1.05	149.16	0.2977	82.84

F8	50	5	2	149.95 ± 5.01	0.2971±0.0011	82.74±1.89	149.16	0.2977	82.84
F9	50	2.5	1	118.07 ± 5.98	0.3568±0.0003	80.04±0.96	117.92	0.3567	80.28
F10	40	5	1	141.34 ± 8.95	0.2445±0.0010	74.50±3.26	141.29	0.2554	74.76
F11	50	2.5	3	123.26 ± 3.40	0.3759±0.0093	81.01±2.61	123.2	0.3775	81.55
F12	50	7.5	1	181.76 ± 6.08	0.2207±0.0050	85.13±3.94	181.1	0.2185	84.85
F13	50	5	2	149.16 ± 4.95	0.2966±0.0008	82.64±0.20	149.16	0.2977	82.84
F14	40	5	3	142.1 ± 3.95	0.2749±0.0012	76.52±2.12	141.91	0.2734	76.74
F15	60	5	3	157.16 ± 4.05	0.333±0.0095	94.71±0.82	157.11	0.3344	94.29
F16	60	2.5	2	129.47 ± 9.61	0.395±0.0006	91.71±1.65	128.99	0.395	91.01
F17	40	2.5	2	112.15 ± 4.03	0.3365±0.0012	72.47±1.23	112.19	0.3379	72.26

Table S2: Regression analysis response summary

Quadratic Model	R^2	Adjusted R^2	Predicted R^2	Adeq Precision	Press	SD	Mean	% CV
Response 1 (Y_1)	1.0000	0.9999	0.9997	571.70	2.40	0.1775	149.43	0.1188
Response 2 (Y_2)	1.0000	0.9999	0.9996	616.42	0.0000	0.0004	2.2967	0.1400
Response 3 (Y_3)	0.9995	0.9988	0.9936	133.45	4.31	0.2230	83.78	0.2661

Vesicle size (Y_1) = $149.16 + 7.85 \times X_1 + 29.01 \times X_2 + 0.0625 \times X_3 - 0.5500 \times X_1 \times X_2 - 0.25.00 \times X_1 \times X_3 - 2.57 \times X_2 \times X_3 + 0.1555 \times X_1^2 + 0.2805 \times X_2^2 + 0.1305 \times X_3^2$

Pdi (Y_2) = $0.2977 + 0.0291 \times X_1 - 0.0691 \times X_2 + 0.0104 \times X_3 + 0.005 \times X_1 \times X_2 + 0.0014 \times X_1 \times X_3 + 0.0000 \times X_2 \times X_3 - 0.0024 \times X_1^2 + 0.0020 \times X_2^2 - 0.0018 \times X_3^2$

% EE (Y3) = $82.84 + 8.60 \times X_1 + 2.81 \times X_2 + 1.16 \times X_3 - 0.7750 \times X_1 \times X_2 + 0.1750 \times X_1 \times X_3 + 0.5275 \times X_2 \times X_3 + 1.12 \times X_1^2 + 0.4937 \times X_2^2 + 0.3938 \times X_3^2$

Table S3: Regression coefficient of different release models for opt-RUT-loaded-GMs

RELEASE MODEL	EQUATION	REGRESSION COEFFICIENT (RUT-loaded-GM)
Korsmeyer Peppas model	$\log (C_o - C_t) = \log K + n \log t$	0.8179

Zero-order	$C_o - C_t = K_t$	0.7571
First order	$\ln C_t = \ln C_o + K_t$	0.7571
**Higuchi model**	$\mathbf{Co - Ct = K^{1/2}}$	**0.9472**
